# Do not lose your patient in translation: Using interpreters effectively in primary care

**DOI:** 10.4102/safp.v65i1.5655

**Published:** 2023-02-27

**Authors:** Talat Habib, Arun Nair, Klaus von Pressentin, Ramprakash Kaswa, Hamid Saeed

**Affiliations:** 1 Department of Family Medicine, Faculty of Health Sciences, University of the Free State, Bloemfontein, South Africa; 2 Department of Family Medicine, Robert Mangaliso Sobukwe Hospital, Kimberley, South Africa; 3 Division of Family Medicine, Faculty of Health Sciences, University of Cape Town, Cape Town, South Africa; 4 Department of Family Medicine and Rural Health, Walter Sisulu University, Mthatha, South Africa; 5 Mthatha General Hospital, Mthatha, South Africa

**Keywords:** medical interpreter, modes of interpretation, types of medical interpreters, cultural liaison, communication barriers, primary care

## Abstract

South Africa is a multicultural society characterised by a rich diversity of languages. As a result, many healthcare providers and their patients often do not speak the same language, which makes communication challenging. The language barriers, when present, require an interpreter to ensure accurate and effective communication between the parties. In addition to assisting in a clear exchange of information, a trained medical interpreter also acts as a cultural liaison. This is especially true when the provider and the patient come from different cultural backgrounds. Based on the patient’s needs, preferences, and available resources, clinicians should select and engage with the most appropriate interpreter. The effective use of an interpreter requires knowledge and skill. Patients and healthcare providers can benefit from several specific behaviours during interpreter-mediated consultations. This review article provides practical tips on when and how to use an interpreter effectively during clinical encounters in primary healthcare settings in South Africa.

## Introduction

With over 50 established and unestablished languages in South Africa,^1^ it is unusual for healthcare providers and patients to share the same first language during most clinical encounters.^2^ Language thus becomes a significant barrier to clear communication between the parties, necessitating the use of an interpreter.^3^ It is a reality that South Africa is a popular destination for cross-border African migrants seeking employment or asylum abroad.^4^ These immigrants often face cultural and linguistic barriers within the healthcare system that prevent them from receiving quality care.^4^ There is growing evidence, locally and globally, that poor communication due to language barriers poses a significant health threat.^5,6,7,8,9,10^ South African legislation recognises the importance of language in healthcare communication and the right of all citizens to receive healthcare and information in a language they are familiar with. This is reflected both in the Constitution and in the *National Health Act (Act 61 of 2003)*.^11,12^ Yet, interpreters are not mentioned in the National Department of Health’s 2030 Human Resources for Health (HRH) Strategy.^13^ Thus, the country has a shortage of health and medical administrative services (Table 13 in the 2030 HRH strategy).^13^

Translation and interpretation are related, yet different skills. While translators deal with converting written text from one language to another, interpreters work with spoken words in live situations like a clinical consultation.^14^ However, interpretation in healthcare involves much more than simply having a bilingual person assist in communication between the patient and the provider.^15^ Trained interpreters improve healthcare quality, clinical outcomes and patient satisfaction.^15,16,17^ To provide holistic and individualised care mandated by the World Health Organization,^18^ clinicians must possess essential knowledge and skills to use interpreters effectively in primary health care.^10,15^

Local studies highlight the negative impact of language barriers on the quality and access to healthcare services and the need for trained interpreters in the South African healthcare context.^5,6,10,19^ In the absence of trained interpreters, primary care providers rely on untrained bi- or multilingual staff, family members, or friends, which presents serious ethical and medical challenges. *Ad hoc* interpreters in local studies were found to have inadequate language proficiency and were prone to frequent errors during interpretation.^5,19,20^ Most published data on this topic comes from the Western Cape Province and isiXhosa-speaking patients.^5^ Thus, a research gap exists regarding other provinces and languages in South Africa. This review article aims to provide clinicians with practical tips on engaging with patients more meaningfully by using interpreters effectively during a consultation. Thus, it will assist in reducing language and cultural barriers within the primary healthcare system in South Africa.

## What is the role of a medical interpreter?

The International Association of Medical Interpreters defines interpretation as:

[*T*]he conversion of a message uttered in a source language into an equivalent message in the target language so that the intended recipient of the message responds to it as if he or she had heard it in the original.^21^

Language is not just a means of expressing our thoughts and ideas; it also carries cultural values, attitudes and identity. As such, an interpreter acts beyond a mere language conduit, becoming a cultural liaison between the patient and the provider.^16^ The Code of Ethics of trained interpreters promotes confidentiality, professionalism and client trust.^22^ Medical interpreters typically use sequential or consecutive interpretation, meaning they speak after the original speaker has finished speaking in the source language.^16^

## When do you need an interpreter?

When a patient and the provider are not proficient in the same language, there is a risk of miscommunication. You must consider the need for interpretation if your patient responds to your questions with a nod or a simple ‘yes’ or ‘no’. An open-ended question that cannot be answered by ‘yes’ or ‘no’ can help determine their proficiency level with the language used. Also, ask them to repeat what you have just said in their own words. This simple test can reasonably indicate whether an interpreter is required.^23^

It is also important to know your own limitations in a particular language. In the authors’ experience, with limited language ability, it is easy to ask questions but difficult to fully understand the patient’s response.

## What are the types of medical interpreters?

- **Professional or trained interpreters:** There is good evidence that a professionally trained interpreter not only improves clinical outcomes and patient satisfaction but also reduces communication errors, unnecessary investigations, hospital stay, readmission rate and malpractice risk.^17,24,25,26^ Their training in language, basic knowledge of medical terminology and diseases, cultural proficiency and professional ethics make them the gold standard. According to the authors’ personal communication with provincial Human Resources officials, except for a few select facilities, most healthcare settings in South Africa lack trained interpreters.
- **Informal or *ad hoc* interpreters:** In the absence of trained interpreters, family members, friends and neighbours accompanying the patient serve as interpreters. They pose serious challenges related to confidentiality, impartiality, increased errors in interpretation, and may distort the message due to cultural reasons or personal agendas.^14,15,16,17,26,27^
  It is important to note that minor children should not be used as interpreters except in emergencies (Box 1).^15,17,29^ Even in unavoidable situations, their use demands careful consideration based on the context of information that needs to be exchanged. There is a difference between using a 10-year-old son to take a menstrual history from his mother and a 16-year-old daughter to enquire about her father’s diabetic medication. Legislations in some countries explicitly ban the use of minor children for interpretation in non-emergency situations.^29,30^
- **Bi- or multilingual healthcare staff:** It includes doctors, nurses, and other staff in healthcare facilities. When trained and appropriately used, they become valuable assets for effective communication. However, errors are more likely to occur because they are often untrained and not formally assessed in their language abilities.^14,16^
  It is common practice in the South African healthcare sector to use untrained bi- or multilingual staff and *ad hoc* interpreters.^20^ Despite not being the first choice, *ad hoc* interpreters (e.g. family or friends) have some benefits and may often be the only option available in the facility (Table 1).

BOX 1 Risks of using children as medical interpreters.

Involving minors in interpretation presents several ethical dilemmas:

- Parents’ privacy can be compromised.
- It can negatively affect family dynamics as it undermines parental authority.
- A child may have poor knowledge of clinical terminology in the source and target languages, increasing the risk of mismanagement.
- During interpretation, exposure to a parent’s serious illness or history of violence and trauma can place a heavy emotional burden on minors.
- To protect their children or to avoid embarrassment, parents may withhold vital information.
- In some cultures, certain topics are considered inappropriate for children.

*Sources*: Please see the full reference list of the article Kasten MJ, Berman AC, Ebright AB, Mitchell JD, Quirindongo-Cedeno O. Interpreters in health care: A concise review for clinicians. Am J Med. 2020;133(4):424–428.e2. https://doi.org/10.1016/j.amjmed.2019.12.008, for more information

**TABLE 1:** Potential benefits and risks of using untrained family and/or friends for healthcare interpretation.

Benefits	Risks
Easy accessibility in resource constraint settings	Language skills may not be optimal Lack of adequate knowledge of medical terminology and diseases
More comfortable for patients and trusted by them	Errors, omissions, and substitutions are more likely to occur
Beneficial in situations where the patient requires advocacy and ongoing support beyond the consultation	No guarantee of confidentiality and privacy Discussions involving sensitive topics are difficult
Ensures continuity during future consultations	Conflict with normal dynamics and roles in the family
Appropriate for less complex clinical scenarios	More tangential conversations interrupt the flow and control of the consultation
In most cases, no financial cost is involved	They may have their own agendas

*Source*: Please see the full reference list of the article Kasten MJ, Berman AC, Ebright AB, Mitchell JD, Quirindongo-Cedeno O. Interpreters in health care: A concise review for clinicians. Am J Med. 2020;133(4):424–428.e2. https://doi.org/10.1016/j.amjmed.2019.12.008, for more information

## What are the modes of interpretation?

These depend on the available resources, for example, in-person, telephonic, video remote interpretation or web-based applications.^24^

- **In-person face-to-face:** The interpreter is physically present with the provider and the patient. It is the preferred choice when available.^14,29^ It enables the user to observe non-verbal language, behaviour and personal characteristics, enhancing communication.
- **Telephonic:** Patients and providers are linked to the trained interpreter via an audio device. It can be a cost-effective solution for remote areas and languages which are less commonly spoken. The patient may remain more anonymous and comfortable, especially when discussing sensitive issues.^15,16^ However, certain limitations, such as loss of body language and a lack of continuity, should be recognised.^5,15,16^ Some developed countries, such as Australia, provide free national telephone interpretation services to their clinicians.^31^ There are few private for-profit companies in South Africa offering professional telephonic interpreter services for a fee.
- **Video remote interpretation:** The use of smart devices and applications to access professional interpretation services is on the rise. With this technology, advanced features, such as spoken words appearing as text on screen, provide an advantage when communicating with a hearing-impaired patient without an in-person sign language interpreter.^15,17^
- **Web-based translation applications:** New applications that enable voice-to-voice and voice-to-text communications are improving. While these applications may be useful in simple, low-risk healthcare encounters, their role in complex healthcare settings has yet to be determined because of multiple patient safety concerns.^28,32,33^

## Medicolegal considerations of working across language and cultural barriers in primary care

If an interpreter is necessary, primary care providers should be aware of the medicolegal risks and implications of not engaging one. It may be crucial in certain situations, such as obtaining a patient’s consent for a procedure or assessing their ability to make decisions.

In its ethical guidance for good practice, the Health Professions Council of South Africa (HPCSA) recommends using an interpreter if a linguistic or cultural barrier prevents effective communication. For instance, HPCSA provides the following recommendation in its guidelines for withholding and withdrawing treatment (booklet 7, p. 4, section 7.1.7):

A linguistic or cultural barrier may exist between health care practitioners and the patient. Under these circumstances, an interpreter fluent in the language used by the patient should be present in order to facilitate communication when discussions are held and decisions regarding the treatment of the patient are to be made.^34^

It is important to understand the potential risks associated with *ad hoc* interpreters when clinical decisions are complex and critical (Table 1).

■ The use of interpreters should be documented. Patients’ clinical and/or medicolegal records should be updated as per HPCSA guidelines (booklet 9).^34^

Currently, there is no explicit policy in the HPCSA guidelines requiring patients to provide written consent if they wish to use language interpretation services.

## How to work effectively and efficiently with medical interpreters?^15,16,17,23,35^

### Before the consultation

- Choose an appropriate interpreter and the mode of interpretation based on specific needs, available resources and patient preference.
- Meet the interpreter to build rapport and share the patient’s background information and expectations during the interview (Box 2)^36,37,38^.
- Schedule the interview for a suitable time and location, allowing extra time.
- Ensure that the equipment for the interview has been tested and is in working order when considering an alternative to in-person interpretation.

BOX 2 Key points on the cultural liaison aspect of interpretation.

- Managing cultural differences is essential when healthcare providers and patients come from different cultural backgrounds.
- Successful intercultural communication involves many factors other than language, such as body gestures, silence, laughter, emotions, etc., varying from culture to culture.
- Interpreters can serve as bridges between cultures.
- Understanding one’s own cultural beliefs, values and practices is essential before learning about other cultures.
- The ability to be sensitive and adaptive to individual cultural differences depends upon the clinician’s self-awareness and reflection.
- Knowledge of a patient’s culturally determined health beliefs and practices assists the clinician in developing a trusting therapeutic relationship.
- Whenever possible, allow the interpreter to offer cultural information.
- It is often helpful to have brief information about the patient’s sociocultural background and expectations. For instance, an interpreter may point out that a patient might avoid handshakes with members of the opposite gender for religious reasons; such behaviour should not be perceived as arrogant or disrespectful.
- The provider should consult the interpreter for clarification when a cultural misunderstanding is suspected.

*Sources*: Please see the full reference list of the article Kasten MJ, Berman AC, Ebright AB, Mitchell JD, Quirindongo-Cedeno O. Interpreters in health care: A concise review for clinicians. Am J Med. 2020;133(4):424–428.e2. https://doi.org/10.1016/j.amjmed.2019.12.008, for more information

### During the consultation

- Introduce and/or identify everyone present during the interview.
- Provide a comfortable environment conducive to the free exchange of information.
- Position the interpreter next to or slightly behind the patient in an ambulatory setting.
- The interpreter and provider should stand side-by-side in a casualty or inpatient setting so the patient can easily see both.
- Be interested and maintain regular eye contact.
- Speak directly to the patient in the first person, using ‘I’ and ‘you’ statements. Do not address the patient indirectly via an interpreter in third-person language, for example, ‘ask him’ or ‘tell her’.
- Speak clearly and slowly, using simple common words and short sentences.
- Limit the number of key points to a minimum and ask one question at a time.
- Avoid slang, complex medical jargon, acronyms and idioms.
- Avoid humour and jokes; they may be difficult to understand or inappropriate in another language or culture.
- Insist on sentence-by-sentence interpretation. The interpreter must not answer for the patient.
- Allow the interpreter sufficient time to answer your question.
- While waiting silently for the patient’s response, use this so-called ‘negative space’ effectively by observing the patient’s non-verbal clues and formulating your next question.
- Stay in command of the interview process. If much side talk takes place, you may interrupt the conversation and regain control.
- Check and reinforce the patient’s understanding by techniques such as ‘teach-back’ or ‘show me’.
- Provide closure at the end of the interview by summarising key information.

### After the consultation

- Thank the interpreter.
- Discuss and clarify medical, social, cultural, or ethical concerns.
- Offer debriefings after emotionally taxing interviews.
- Plan follow-up appointments and referrals to the primary care team members or other levels of care if indicated.
- The use of an interpreter should be documented in the patient’s clinical notes and/or medicolegal records.

## Recommendations

Medical interpreters are an important and relevant staffing category within primary healthcare. Their role and value in healthcare teams should be recognised within national HRH policy to address the current gap. This will ensure that primary care teams comprise appropriate human resources and skill sets.

Because of the risks associated with *ad hoc* interpreter use, there is an urgent need to establish easily accessible trained interpreter services in the primary health care sector in South Africa. The availability of trained interpreters can be further supported by a centralised telephone interpreter service.

Education modules on the use of interpreters may be incorporated into undergraduate, postgraduate or registrar training and continuing medical education programmes. Courses designed to enhance communication skills and use interpreters effectively should be offered to primary care providers. Additionally, language courses might be offered at the undergraduate and graduate levels of health professions education.

Bi- or multilingual medical staff, with adequate training and support, can serve as valuable backup resources.

## Conclusion

Patients with limited proficiency in the provider’s language need interpreters. Effective use of interpreters requires knowledge and skill sets. Interpreters are valuable communication aids and cross-cultural guides in healthcare. Type and mode of interpretation depend on the need of the patient, availability of resources and patient preference. A trained and professional interpreter is the gold standard. Informal *ad hoc* interpreters should be used with caution.

Minor children should not be used as interpreters except in an emergency. During the consultation, speak directly to the patient using first-person language. Insist on sentence-by-sentence interpretation, observe non-verbal clues and stay in control of the process. Be sensitive to cultural differences.
